# Studies on In Vitro Antimicrobial, Anticancer, and Anti-oxidative Inflammatory Response of Methanolic Tuber Extracts Derived From Terminalia chebula

**DOI:** 10.7759/cureus.63930

**Published:** 2024-07-05

**Authors:** Devadharshini Suresh kumar, Arockia Alex, Brahma Neha, Kalpana R, Sugumar Vimal

**Affiliations:** 1 Department of Biochemistry, Saveetha Medical College and Hospital, Saveetha Institute of Medical and Technical Sciences (SIMATS), Chennai, IND; 2 Department of Bioengineering, Saveetha School of Engineering, Saveetha Institute of Medical and Technical Sciences (SIMATS), Chennai, IND

**Keywords:** antimicrobial activity, in-vitro anti-inflammatory activity, anti-oxidative effect, anticancer activity, terminalia chebula, oral cancer cell line (kb-1)

## Abstract

Aim

This study aims to investigate the antibacterial, antifungal, and phytochemical properties of methanolic tuber extracts from *Terminalia chebula*. Additionally, the study seeks to assess the in vitro anticancer effects of these extracts on an oral cancer cell line, as well as their antioxidant and anti-inflammatory activities.

Materials and methods

The research involves examining the antibacterial and antifungal properties of methanolic tuber extracts from *Terminalia chebula*. The phytochemical composition will be analyzed using standard techniques. The in vitro anticancer effects will be tested on an oral cancer cell line, while antioxidant and anti-inflammatory activities will be evaluated through appropriate assays.

Results

The study demonstrated that *Terminalia chebula* methanolic tuber extracts exhibit cytotoxic effects on the oral cancer cell line (KB-1), reducing cell viability as evidenced by the 3-(4,5-dimethylthiazol-2-yl)-2,5-diphenyltetrazolium bromide (MTT) assay. A concentration of 30 µg/mL induced notable morphological changes observed under an inverted fluorescence microscope. Antioxidant assays showed a maximum absorption of 85.3% with 50 µL of the extract, while anti-inflammatory tests revealed a 76.0% absorption. Antimicrobial activity, assessed via agar-well diffusion, indicated significant antibacterial effects, especially against *Streptococcus mutans* and *Candida albicans* at higher concentrations. The findings suggest promising therapeutic potential for *Terminalia chebula* extracts.

Conclusion

*Terminalia chebula* tuber extracts may treat diseases caused by studied organisms. The study suggests that methanolic extracts from *Terminalia chebula* tubers have potential commercial value due to their anti-inflammatory, antioxidant, and cytotoxic properties. The extracts induced apoptosis in an oral cancer cell line at 30 µg/mL after 24 hours. Further research is needed to understand the active components and underlying molecular mechanisms.

## Introduction

According to the World Health Organization, it is reported that for primary healthcare about 80% of the world's population relies on plant-based traditional medicines. [1]. During the examination of several medicinal plants, scientists identified *Terminalia chebula* Retz. (Combretaceae) as one of the highly esteemed medicinal plants [2]. This plant demonstrated multiple medicinal activities owing to the abundance of various types of phytoconstituents. The tree's fruit provides a wide range of health benefits and has been employed as a traditional household remedy for addressing various human ailments since ancient times [3,4]. *Terminalia chebula*, commonly called a black myrobalan, belongs to the family Combretaceae. It has been widely utilized in Ayurveda, Unani, and Homoeopathic medicine, gaining significant attention in modern medicine. It is globally distributed, mainly in the subtropical and tropical regions of Asia. In India, it is found in the forests of Northern India, Southern Maharashtra, Uttar Pradesh, West Bengal, Tamil Nadu, and Karnataka. It is the second-largest genus in the family, with nearly 200 species of Terminalia species. Dash and Bhagwan (1991) reported that in Tamil Nadu, tribal communities commonly employ *Terminalia chebula* as a traditional remedy for addressing various health issues such as fever, cough, diarrhea, gastroenteritis, skin disorders, candidiasis, urinary tract infections, and wound infections [5]. It was used for the treatment of respiratory ailments such as respiratory tract infections and served as a protective measure for the liver [2]. Over the past few years, there has been a surge in bacterial resistance to numerous traditional antibiotics, leading to the emergence of extremely drug-resistant (XDR) or totally drug-resistant (TDR) strains in several significant bacterial pathogens [6]. Extracts from *Terminalia chebula* gall have shown properties such as antioxidant, anti-inflammatory, antibacterial, anti-arthritic, and anti-aging [7-9]. Apart from their effectiveness in treating diarrhea, extracts from plant components are also employed for the treatment of tuberculosis and cough [10]. While cancer treatments aim to destroy cancer cells, they may inadvertently damage healthy cells, leading to various side effects [11,12]. A previous investigation indicated that a 70% methanol extract derived from *Terminalia chebula* fruits exhibited efficacy in neutralizing free radicals [13].* Terminalia chebula* demonstrates superior antibacterial activity compared to *Acalypha indica* [14]. The 3-(4,5-dimethylthiazol-2-yl)-2,5-diphenyltetrazolium bromide (MTT) assay showed a dose-dependent percentage inhibition. Evaluation of cytotoxicity for doxorubicin and *Acalypha indica* revealed a decrease in percentage viability with increasing concentration. A 4′,6-diamidino-2-phenylindole (DAPI) staining indicates that apoptosis reaches its peak at the 24th hour [15]. 

## Materials and methods

Plant extract collection and preparation

Dried fruits of* Terminalia chebula* were procured from a local market in Chennai, India, and subsequently prepared for extraction. After thorough cleaning and drying, the fruits were finely powdered to facilitate the extraction of their active compounds. The powdered plant material was then mixed with an appropriate solvent, such as methanol, and subjected to extraction using methods like maceration, sonication, or Soxhlet extraction [16]. The resulting methanolic extract was obtained, and the supernatant was carefully collected and filtered through Whatman No.1 filter paper to remove any solid residues. The extract was then concentrated using a rotary flask evaporator under controlled temperature conditions determined by the properties of the solvent system employed. This process aimed to obtain a concentrated extract rich in the active compounds present in *Terminalia chebula* fruits, suitable for further analysis and research purposes.

Oral pathogen study

Test pathogens were maintained on Muller-Hinton agar (MHA) as a stock culture, initially recovered from swab sticks. To assess the antibacterial activity of the methanolic extract of *Terminalia chebula*, the MHA medium was utilized through the well diffusion method. The methanolic extract demonstrated antimicrobial properties [6]. Using a sterile cotton swab, 16-hour pathogens were evenly distributed on the surface of MHA. After a 10-minute incubation, wells were created in the agar medium using acorkborer. The MHA medium underwent sterilization at 121°C and 15 pounds of pressure for 20 minutes. For antibacterial and antifungal activity, the resulting dry extract was dissolved in dimethyl sulfoxide (DMSO) or distilled water to create a stock solution [8]. The methanolic extract of *Terminalia chebula* was made in 20% DMSO and put into four separate wells. The concentrations were 25 g/mL, 50g/mL, and 100 g/mL. A negative control containing 20% DMSO was also included. Following 24 hours of incubation at 37°C, the zone of inhibition was measured to evaluate antimicrobial effects. A group of supernatants was collected, and the extraction process was sped up with Whatman No. 1 filter paper and a rotary flask evaporator at a temperature that depended on the solvent system. The zone of inhibition was measured after 24 hours of incubating at 37ºC.

Cell line maintenance

Cancer cell lines (KB-1) were cultured in a suitable medium with 10% FBS and a 1% penicillin-streptomycin solution. The cells were kept at 37°C in a 5% CO_2_ incubator.

Cell viability (MTT assay)

The MTT assay was used to assess the viability of KB-1 cells treated with *Terminalia chebula* extract [17]. The following formula was used to determine cell viability: (A570 nm of treated cells/A570 nm of control cells)x100=% cell viability.

Morphology study

Optimal dosages (IC-50: 30 µg/mL) determined from the MTT assay were used for further study. The morphology of KB-1 cells was observed under a phase-contrast microscope after introducing *Terminalia chebula* (30 µg/mL).

Determination of mode of cell death

The cell's monolayer was fixed with 3% paraformaldehyde and cleaned with phosphate-buffered saline (PBS) for nuclear morphological examination. A fluorescent microscope was used to study apostrophic nuclei, which are defined by highly colored, fractured nuclei and compressed chromatin [18].

Statistical analysis

Data, presented as mean±SD for triplicates, underwent one-way ANOVA analysis in SPSS before a student's t-test.

In vitro antioxidant-H_2_O_2_ scavenging activity

A technique described was used to assess the H_2_O_2_ scavenging capability of the methanolic *Terminalia chebula* tuber extracts. Using the formula radical scavenging activity (%)=(Abs control minus Abs sample/Abs control)×100 the percentage of H_2_O_2 _radical scavenging was determined. The reaction mixture (10 mL) contained 1 mL of the test extract and reference medicine at five different concentrations (10 mL, 20 mL, 30 mL, 40 mL, and 50 mL), 3 mL of PBS (pH: 6.4), and 1 mL of each of the other [19]. 

In vitro anti-inflammatory activity 

The reaction mixture (10 mL) contained 1 mL of the test extract and reference medicine at five different concentrations (10 mL, 20 mL, 30 mL, 40 mL, and 50 mL), 3 mL of PBS (pH: 6.4), and 1 mL of egg albumin (EA) (1 mM). With Vt representing the test sample's absorbance and Vc representing the control sample's absorbance, the formula for calculating the percentage inhibition of protein denaturation is % inhibition=100 times (Vt/Vc-1), where Vt=absorbance of the test sample and Vc=absorbance of control.

## Results

**Figure 1:**
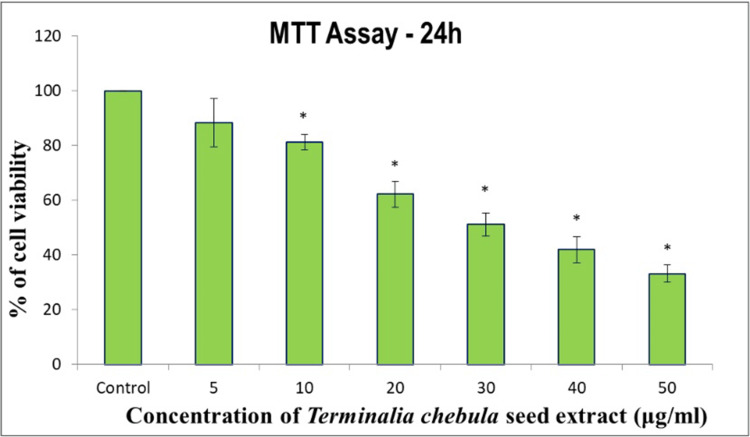
In vitro anticancer activity of methanolic extract of Terminalia chebula on oral cancer (KB-1) cell line. Data are shown as means±SD (n=3) ^*^compared with the control blank group, p<0.001.

The cytotoxic impact of *Terminalia chebula* on the oral cancer cell line (KB-1) was assessed through a 24 hour treatment with varying concentrations (5-50 μg/mL), and cell viability was measured using the MTT assay, which is shown in Figure 1. The MTT assay data underlie the potential cytotoxic effects of *Terminalia chebula* on the oral cancer cell line (KB-1), suggesting its capacity to influence cell survival and further exploration for potential therapeutic applications or as a basis for drug development.

**Figure 2:**
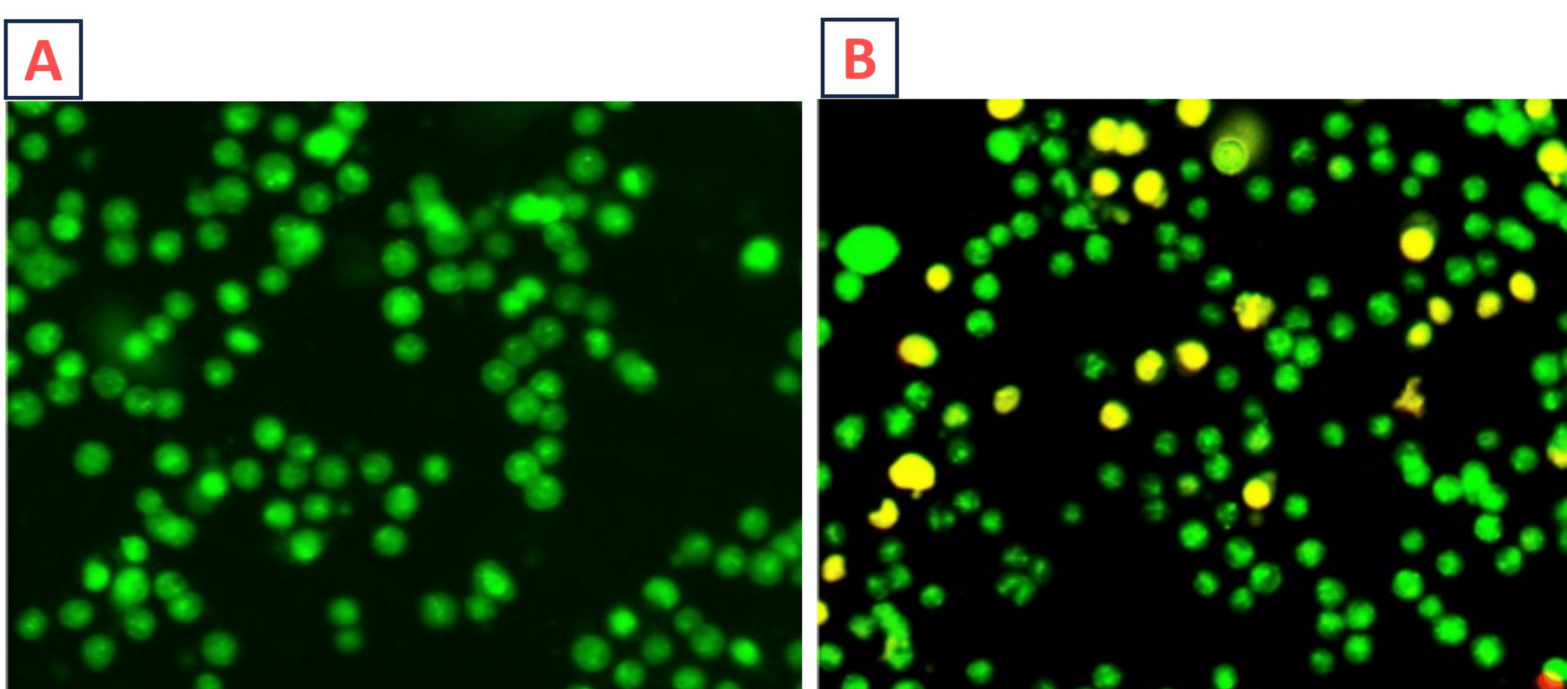
(A) Control group (without treatment) and (B) cells were treated with Terminalia chebula methanolic extract (30 µg/mL) for 24 hours along with the control group. Images were obtained using an inverted fluorescence microscope

Cells were exposed to a 24 hour treatment with a 30 µg/mL concentration of *Terminalia chebula* methanolic extract in comparison to a control group. Subsequent to the treatment, cellular responses were visualized through an inverted fluorescence microscope, as shown in Figure 2. This microscopic analysis aimed to unveil any morphological or fluorescence changes induced by the *Terminalia chebula* extract. The choice of the methanolic extract and the specified concentration suggest a focus on potential bioactive compounds within the plant, emphasizing the exploration of cellular interactions and alterations.

**Figure 3:**
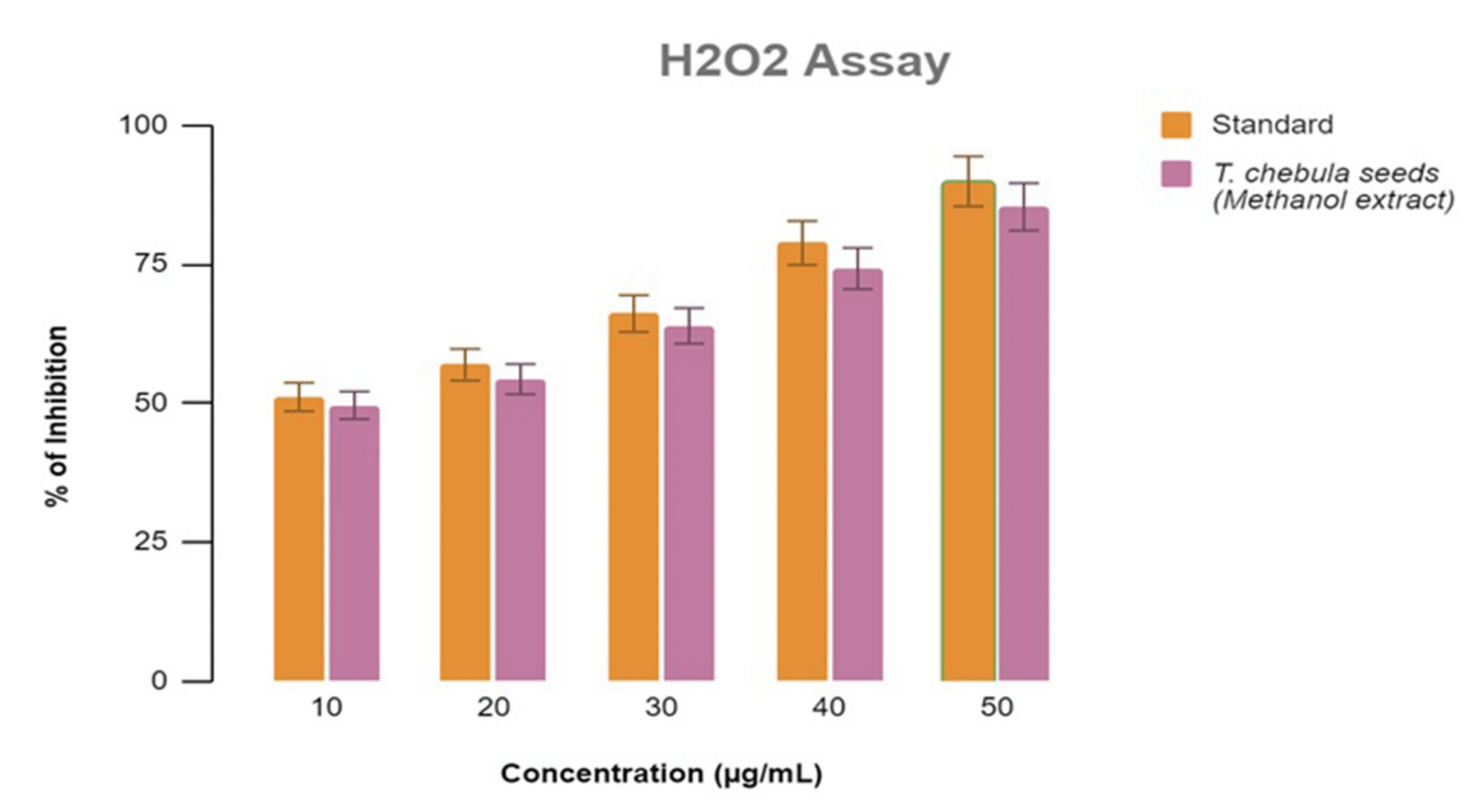
In vitro antioxidant assay revealed that 50 μL of the methanolic tuber extracts of Terminalia chebula had the highest absorption percentage of about 85.3%. Ascorbic acid was used as the standard reference for this assay

**Figure 4:**
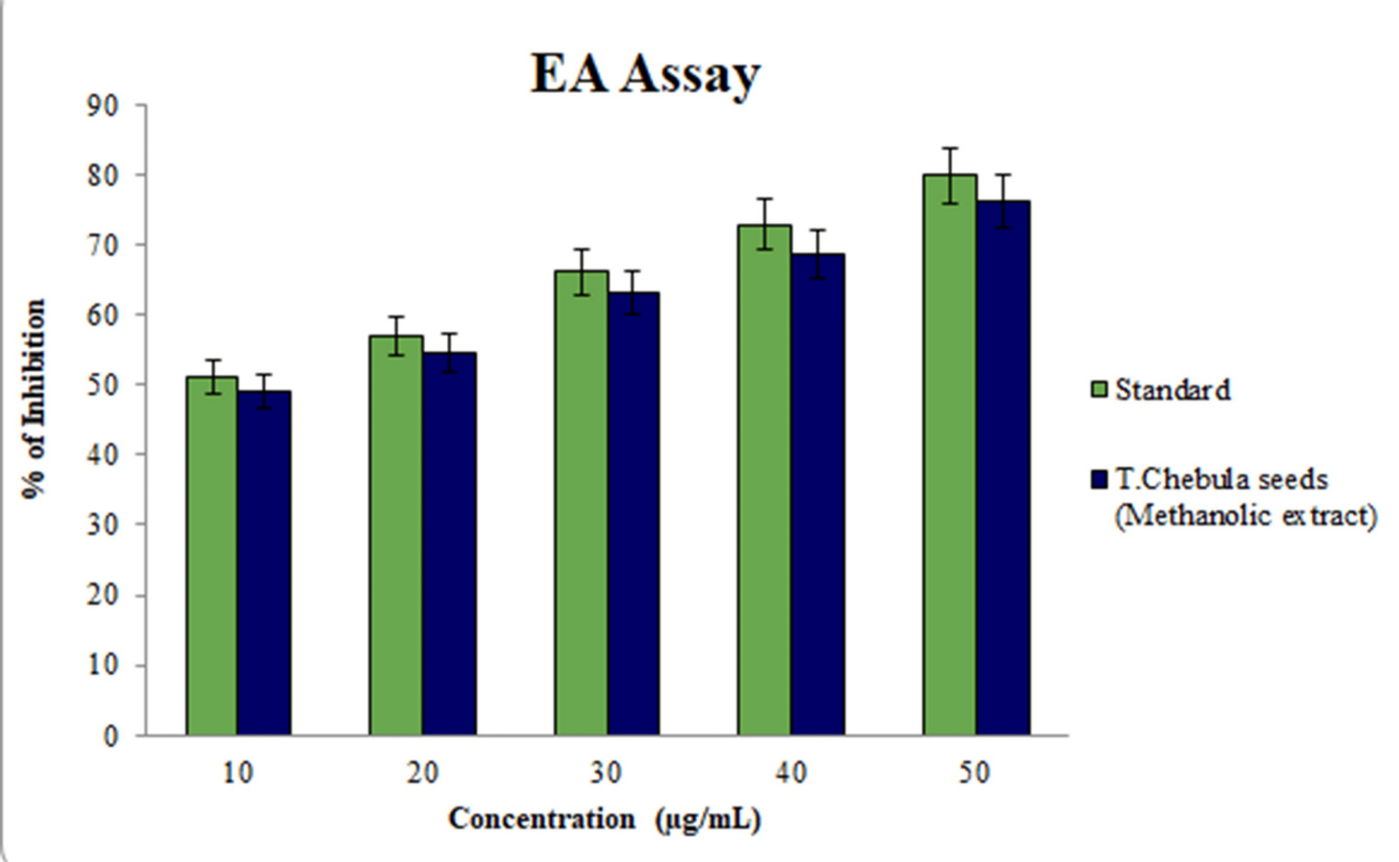
In vitro anti-inflammatory assay revealed that 50 μL has an absorption percentage of about 76.0%. Diclofenac was used as the standard reference for this assay

Using a spectrometer, the antioxidant assay findings revealed that the maximum absorption percentage was achieved by 50 μL of methanolic tuber extracts from *Terminalia chebula*, reaching an astonishing 85.3% in Figure 3. In the meantime, 50 μL of the tested sample had an absorption percentage of around 76.0% in Figure 4, according to the anti-inflammatory testing findings. Thus, the study showed that the methanolic extract of *Terminalia chebula* had a greater percentage of inhibition in both antioxidant and anti-inflammatory test analyses.

**Figure 5:**
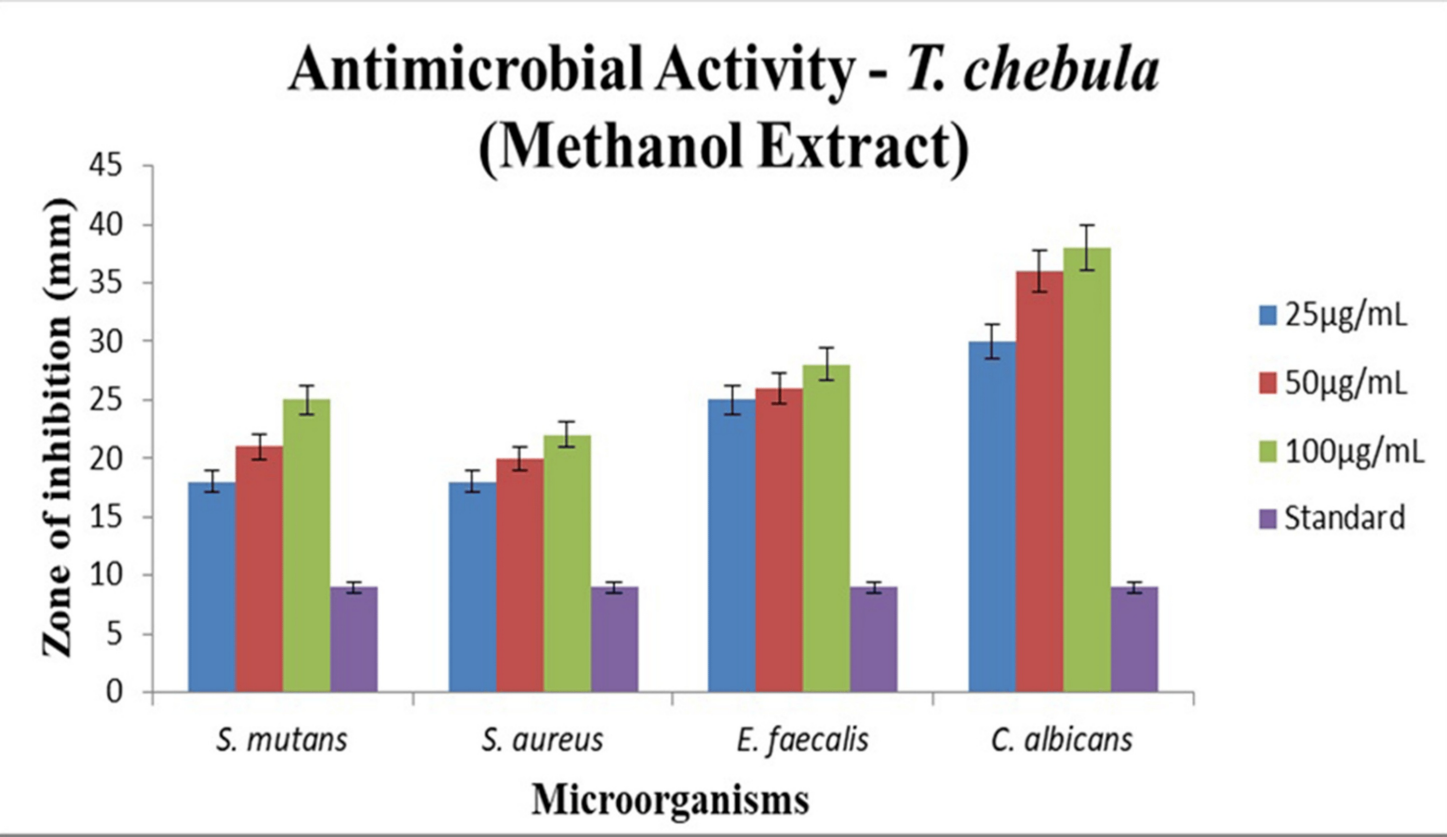
In vitro antimicrobial activity of Terminalia chebula by agar-well diffusion method. DMSO was used as the standard reference for this assay DMSO: dimethyl sulfoxide

The agar-well diffusion technique, a widely used methodology for evaluating antimicrobial activity, was applied in the study. The study's objective was to look at the blocking zones that formed around the wells made in an agar medium after the methanolic extract of *Terminalia chebula* was added. This would shed light on the extract's possible antibacterial activities, as seen in Figure 5. The study observed that *Candida albicans* exhibited strong antimicrobial activity solely at higher concentrations (100 μg/mL), with no effect at lower concentrations. *Streptococcus mutans* exhibits higher antimicrobial activity compared to the other bacteria tested. Above all, *Candida albicans* shows a high antimicrobial effect, with activity concentrations increasing, as shown in Figure 5.

## Discussion

The present study identified antimicrobial, anticancer, and anti-oxidative inflammatory responses of methanolic tuber extracts derived from *Terminalia chebula*. This study aimed to explore the antioxidant, anti-inflammatory, antibacterial, antifungal, and cytotoxic properties of methanolic tuber extracts from *Terminalia chebula* on an oral cancer cell line. The findings align with previous research indicating the medicinal potential of *Terminalia chebula* due to its rich phytochemical composition. The antioxidant capacity of *Terminalia chebula* methanolic extracts was evident from the high absorption rate of 85.3% observed in the antioxidant assay, attributable to the presence of phenolic compounds, flavonoids, and vitamins known to neutralize free radicals and reduce oxidative stress [2,7,13]. The anti-inflammatory activity was significant, with a 76.0% absorption rate, aligning with prior studies demonstrating the anti-inflammatory properties of *Terminalia chebula* [8]. Antibacterial and antifungal activities were notable, particularly against *Streptococcus mutans* and *Candida albicans*, respectively, confirming the antimicrobial efficacy reported in earlier research [9,14]. The cytotoxic effects on the oral cancer cell line (KB-1) were substantial, with 30 µg/mL inducing significant morphological changes, supporting the potential therapeutic applications of *Terminalia chebula* in cancer treatment [14,20]. These findings suggest that *Terminalia chebula* methanolic tuber extracts possess considerable antioxidant, anti-inflammatory, antibacterial, antifungal, and cytotoxic properties, warranting further investigation into their active components and mechanisms of action.

## Conclusions

The study suggests that methanolic extracts from *Terminalia chebula* tubers might be used as an alternative to commercial value and may possess anti-inflammatory and antioxidant characteristics. The study results indicated that plant extracts from *Terminalia chebula* were cytotoxic and induced apoptosis in the oral cancer cell line at a dosage of 30 μg/mL after 24 hours of incubation. Additional research is needed to fully comprehend the active components extracted and the molecular processes responsible for their anticancer properties.
